# Perceptions of diabetic retinopathy patients on the management of diabetic retinopathy (DR) in South Tarawa, Kiribati: A qualitative study

**DOI:** 10.1371/journal.pgph.0004103

**Published:** 2025-01-21

**Authors:** Teuota Angirerei-Farran, Masoud Mohammadnezhad, Kal Alnababatah, Mosese Salusalu

**Affiliations:** 1 Department of Public Health, Ministry of Health & Medical Services, Nawerewere, Tarawa, Kiribati; 2 School of Nursing and Midwifery, Birmingham City University, Birmingham, United Kingdom; 3 Department of Health Education and Behavioral Sciences, Faculty of Public Health, Mahidol University, Nakhon Pathom, Thailand; 4 Department of Public Health, Daffodil International University (DIU), Dhaka, Bangladesh; 5 School of Public Health and Primary Care, Fiji National University, Suva, Fiji; PLOS: Public Library of Science, UNITED STATES OF AMERICA

## Abstract

Diabetic Retinopathy (DR) is one of the most common causes of legal blindness in developing countries, particularly between the ages of 20 to 65 years. Kiribati is currently facing the burden of DR where more than 5% of diabetes patients had experienced negative impacts of DR. This study aimed to explore the perceptions of DR patients on DR management in South Tarawa, Kiribati. This qualitative study was carried out at the Eye clinic in Tarawa Central Hospital from the 29^th^ of August to 23^rd^ of September, 2022. Patients diagnosed with DR of both sexes aged ≥ 18 years were purposively selected to participate in this study. 27 DR patients were recruited and interviewed using a semi-structured open-ended questionnaire. Manual thematic analysis was applied to observe the similarities and differences in answers obtained from interview transcripts. A total of 27 DR patients were enrolled in this study. The majority of patients were between the age of 50–59 (37%) and were males (62%). The findings highlighted a lack of knowledge and awareness of DR management among patients with diabetes in Kiribati. Poor health education, in-availability and lack of access to eye care services, patient belief, and healthcare system issues were identified as the most crucial contributing barriers. These data characterized the need for more communication campaigns including specific messaging on DR and its management to increase diabetes patients’ awareness of the importance of DR treatments.

## Introduction

Diabetic Retinopathy (DR) is a complication of Diabetes Mellitus (DM) that causes preventable blindness among individuals with diabetes and is commonly found in people aged 20 to 65 years, particularly in low-middle-income countries [1,2]. Globally, 2.5 million of people were having visual impairment due to DR in 2015 and the number was increasing to 3.2 million in 2020 [2]. The prevalence of DR ranges from 10% to 50%, depending on the population and methods used to screen for DR and the duration of diabetes [3]. The incidence of DR in the Pacific countries was higher compared to developed countries because of their limited resources and lower income to improve DR therapies management [4]. There are however, factors that influenced DR progressions such as blood pressure, blood glucose, and serum lipids [5]. DR also has a significant impact on quality of life, particularly for patients in advanced stages, who experience negative effects such as reduced self-dependence, loss of employment, lack of social participation, and depression [6,7].

DR is a health issue affecting adults of working age globally [8]. Epidemiology studies confirmed that patients with diabetes can experience and develop DR mostly after 20 years [9]. Since, DR is the leading cause of preventable blindness globally, it is crucial to concentrate more on these target groups as they found to be the most critical to behavior changes and lifestyle which dramatically increased the incidence of Type 2 DM (T2DM) which can led to DR blindness. In addition, these target groups are also working adults that can be successful at work to meet their family needs and as well as the country.

DR blindness is an important health issue to be considered because it may strain the country’s economy, particularly in Lower- Middle-Income Countries (LMICs). Previous studies commented that a large amount of money was spent on treatments for DR [5,10,11]. For example, in Sweden, the annual average healthcare cost of any DR, Proliferative Diabetic Retinopathy (PDR), and Diabetic Macular Edema (DME) amounts to USD$93.6, USD$334.1, and USD$280.8, respectively [12]. However, proper interventions on DR management were crucial to reduce DR blindness and to prevent economy lost from purchasing of expensive treatments and equipment. Since vision loss is not present in the first stage many studies stated that DR screening is very crucial for DR patients because DR is asymptomatic and patients could not notice it presence until the last stage which is not effective to treat (Day et al., 2019; Wykoff et al., 2021). Blindness from diabetic retinopathy (DR) can be reduced with routine eye screening and prompt treatment. A proper health education on diabetes and DR increase patient’s adherence to their DR treatments and compliance to DR screening.

DM has also found high in the Pacific regions compared to the global prevalence of 8.5%. This indicated that the incidence of DR has been on the rise in the Pacific as well [10]. In Kiribati, diabetes was life threatening and bring a double burden to the healthcare system [13]. The World Health Organisation (WHO) highlighted that Kiribati is positioned number 2 in the world for its higher number of deaths due to DM (153 or 12.80% of the total deaths with an adjusted death rate of 208.69 per 100,000 per population) [14]. There are more than 25% of the adult population has received treatment for diabetes and pre-diabetes, and 100 new cases diagnosed each year [15,16]. increasing trends towards, obesity and overweight, physical inactivity and wrong diets are strongly associated with risk of diabetes and DR in Kiribati.

Though DR is found true in Kiribati with more than 1000 of diabetes patients diagnosed and got visual impairment [13], understanding of the disease is not very common and this may due with the lack of proper study that particularly focuses on the perception of patients on diabetes management. While DR is known as the main cause of new cases of blindness in middle age and elderly, there is limited studies done in the pacific countries on DR, hence this study aimed to explore the perception of DR patients on the management of DR in South Tarawa, Kiribati. Collection of patient’s perceptions on DR management is crucial to improve on diabetic and DR management such as implementing of new strategies and interventions that could tackle DR blindness in the pacific and worldwide. This study will also increase DM patients’ awareness on DR to increase their presentations to their DR treatment therapy and Eye checkups.

## Methods

### Study design and setting

This study applied a descriptive qualitative method to examine the perceptions of DR patients on the management of DR. A qualitative study provides a factual response to questions on how the people feel about a certain situation. It also consents to an in-depth exploration of respondent’s attitudes, experiences and intentions on the topic (Lambert & Lambert, 2013). The interview with the participants was conducted from 29^th^ of August to 23^rd^ of September, 2022. This study was carried out at the Eye clinic in Tarawa Central Hospital as it was the main referral hospital in Kiribati. Kiribati is one of the most remote and dispersed places in the world, so the transportation and telecommunication are time consuming, difficult and expensive. There is only one small eye clinic led by a fully trained ophthalmologist which delivered eye care for the entire country, which is dealing with approximately 100 diabetes patients who need to be screened for signs of DR every month. This clinic is capable with sorts of sight-restoring procedures being performed every Monday in the theatre and abiding clinics throughout the week for diagnosis and non-surgical treatment.

### Study sample

Twenty-seven DR patients were interviewed until data saturation was achieved and there was no new information obtained through the interviews. Patients were classified as DR when they diagnosed with DR by professional eye care professionals. A purposive sampling method was employed to recruit the participants across gender, geographical locations, DR stages, years since DR diagnosis, and patterns of attendance, experiences, and opinions. Purposive sampling utilized based on the characteristics of the participants studied and because of the limited number of people who could serve as a primary data source [17]. The inclusion criteria for this study include, DR patients who consented to participate in the study, male and female over the age of 18 years, and they were current patients at the eye clinic in South Tarawa. The exclusion criteria included; patients with cognitive impairment and those who were not interested in this study.

### Data collection tool

Semi-structured open-ended questionnaires were used to guide an in-depth interview with patients. Interview questions are based on the study research questions and the aim. The collection of data involved structured questionnaires on participant characteristics such as the age, gender, DR stage (Mild: with few microaneurysms, Moderate: with increased number of microaneurysms and dot-blot hemorrhages, and Severe: “4-2-1 rule” [14]. There was also seven open-ended questionnaires. Questionnaires were all written in English and translated into Kiribati language to explore patients’ experiences on DR management. A semi-structured open-ended interview guide was pre-tested among a small sample of patients in the Eye clinic before the main procedure. In accordance to Balasopoulou (2017), the pilot study was the first step of the entire research protocol, assisting in the planning, and modification of the main study [18].

### Study procedure

After ethical approval and permissions sought from the Ministry of Health in Kiribati, the main researcher who has been trained, contacted the study participants through flyers two weeks before the interview started. Information sheets which contain information on the main purpose of the study and many other relevant information were translated to Kiribati language and distributed among all participant who were recruited for the study. Informed consent was also obtained from participants prior the interview conducted.

### Data analysis

The main researcher coded all interview transcripts and the codes were checked by co-researchers to ensure triangulation in analysis. Interviews were first transcribed into the Kiribati language and then translated into English version. Manual thematic analysis was applied to analysis the data. As Kiger and Varpio (2020) mentioned that thematic analysis is a flexible method of describing data involving six steps such as, familiarization with the data, coding of data, looking for and reviewing data themes, defining and naming these themes, and generating the final report [19]. We began by noting preliminary ideas for codes that could describe the interview content and address the research questions. Then, we manually coded the data using different numbers. Sticky notes and a whiteboard were used to organize similar codes by color, which helped the researcher clearly identify and group the themes. Final themes and subthemes were generated and make up the result of this study.

### Study rigor

This study ensured that criteria of trustworthy was followed in accordance with Guba and Lincolin (1989) proposed. To achieve transferability of this study, the main researcher was using a purposive sampling method to recruit participants from range of ages, locations and socio-economic status to be able to generalize the results to whole population in Kiribati. The data collection was continued until data saturation was obtained. Credibility was enhanced by carrying out a pilot interview among interviewers to ensure feasibility of the study. The main researcher who has been taught and qualified was the one who conducted this research. In this study, dependability was ensured through thorough documentation of the research process, along with replications and verifications. An appropriate study design was selected, and the interview questions were developed using a pilot study. The interviewer was supported by a trained and qualified nurses. A systematic approach was applied throughout the entire process, including the selection of students and study participants, conducting interviews, translation, code selection, generating themes and sub-themes, and reviewing the accuracy of the documentation and interview records. Regarding the reflectivity, this research involved a team of researchers with diverse backgrounds, gender, and levels of experience. The principal researcher, a qualified nurse working at a diabetic retinopathy center in Kiribati, was also trained in conducting qualitative studies. She had already discussed the topic with both colleagues and patients, providing sufficient information to help select and involve patients in the study. She also ensured that a suitable setting was arranged for conducting interviews. The entire process was reviewed by co-researchers, all of whom were experienced in qualitative research. Additionally, the codes extracted from the interviews, the transcripts, and the translations—performed by a bilingual translator—were thoroughly.

### Ethical consideration

The study protocol approved by the College Health Research Ethics Committee (CHREC) at the Fiji National University (FNU) with the ID# 096.22 and the study was conducted in accordance with the tenets of the declaration of the Ministry of Health in Kiribati. Formal written consent was obtained from all participants before enrolling them in the study. Participants were also assured of their identity confidentiality and their details were coded into numerical data. The transcriptions and forms obtained from this study were kept in a safe and locked place and will destroy after two years.

### Inclusivity in global research

Additional information regarding the ethical, cultural, and scientific considerations specific to inclusivity in global research is included in the Supporting Information (S1 Checklist).

## Results

### Characteristics of participants

All the study participant recruited for this study contributed, with none declining to participate so 27 DR patients were interviewed for the study. As Table 1 reveals, the majority of patients were between the ages of 50–59 years (37%) and were males (62%), moderate DR (56%), severe DR (41%) and mild DR (3%). The Majority of patients were attending secondary level (67%), patients with 11 years onset of DM (63%), patients with more than 6 years duration of visual impairment (70.4%).

**Table 1:** Demographics information.

Variables	Frequency	Percentage
**Gender**		
Female	10	37
Male	17	62
**Age**		
40–49	8	30
50–59	10	37
60–69	8	30
70–79	1	3
**DR Stage**		
Mild	1	3
Moderate	15	56
Severe	11	41
**Education background**		
Primary	7	26
Secondary	18	67
Tertiary	2	7
**Years with DM**		
≤5 yrs	2	7
6–10 yrs	8	30
≥11 yrs	17	63
**Duration with VI**		
>1 yr		
1–5 yrs	1	4
6–10 yrs	19	70.4
>10 yrs	7	26

### Themes and subthemes

There are four themes excerpts from data analysis including awareness of DR, perceptions toward DR management, perceived barriers and challenges to diabetic eye care, and suggestions to improve DR management (Table 2). The participant’s identity was coded using letters and numbers such as, PM1, or PF1 which means; PM1 stands for participant male number 1 and PF1 stands for participant female number 1 and so on.

**Table 2:** Main themes from DR patient’s in-depth interview.

Themes	Sub-themes	Open codes
Awareness on DR	Knowledge and concern about DR. Symptom experiences and factors influence DR. Possible impacts of DR	*it affected the whole parts of the body, I need someone to drive me, Diabetes was affected me so much, We couldn’t support our family anymore.*
Perceptions toward DR management	Believe about DR treatment. Herbal medicines Doctor-patient relationship	*I am aware that it has treatments, I did not know what the laser and injection, Specialized doctors can help with retinopathy diseases, I feel that injection was better, Injections helped me a lots compared to laser, The laser helped my vision to stable but I was scared of it, I used the non-juice for my vitamins, The feedbacks from the doctor encouraged me, Sometimes doctors were not talked nicely.*
Perceived barriers and challenges to diabetic eye care	Education and knowledge about DR Health care system factors Access to the services	*I cannot remember because the words are complicated, Most of the time I have waited for the bus, It cost me a lots to access the eyecare services, I don’t have my folder, It took time for the nurse to obtain the doctor’s consent for my travel, Medication out of stock was affected us.*
Suggestions to improve DR management	Resources Advanced treatments and skilled personnel Awareness	*Government should support more trainings for HCWs, I had heard, that there are doctors in overseas which are more specialized, I became an advocator for DR in my work place*

#### Theme 1: Awareness on DR.

This theme includes three sub-themes including the knowledge and concern about DR, symptom experiences and factors influence DR, and possible impacts of DR.

***Knowledge and concern about DR*:** Almost of the patients involved in this study were familiar with diabetes however, understanding about DR was not a common thing even though they were living with the disease for many years. According to 4 patients, they understood that DR is related with diabetes and they aware that diabetes could affect the eyes. Others reported that they have heard about DR but they only concerned about their kidney. One male patient says.

*“Diabetes is the disease that we should not joke about, it affected the whole parts of the body, firstly my legs were amputated and now my eyes were going to blind.... I encouraged my friends who have diabetes to control their blood sugar otherwise they will end up like me”. (PM1, 50 old)*

However, many patients mentioned, that they just found out about DR on their first day of diagnosis but they did not know the cause and even the word retinopathy. They conveyed that they just got DR very sudden. Some cited that they came for other eye problem and they found out to have DR. Few patients reported that they were aware of DR from the doctor when they admitted to the hospital, while several patients voiced that they got the disease very sudden. One patient shared her experienced.

*“It was very frustrated.... because there is no signal telling you that you should go and check your eyes. I was very surprised when I woke up and my left eye was black out, it like a dark cloud covered......till now I am still frustrated....” (PF9, 52 yrs old)*

The majority of patients were realized that DR could lead to blindness however, patients have perceived different levels of sight threatening and emotions, such as patients with severe and moderated DR stages were more high risk to vision loss compared to patients with mild stages. Some patients were depressed and they came with negative thinking. Notwithstanding, patient with mild stages of DR were sound very peaceful.

*“When the doctor told me that I got severe DR, I was so sad......cried.......because someday I will not see my children. I always recall my doctor word...that few more time I will totally blind.... I asked God to help me .... the doctor also told me to control my diabetes (PF1,43 yrs old)*

Overall, most of the participants with moderate and severe stages of DR were more adhered to their treatment therapy and follow up review because they don’t want to lose their vision. They perceived that vision loss is the most difficult complication of DR. One patient responded;

*“I attended the clinic since 2018, for cataract surgery and followed by laser therapy and injection. I was committed because I was afraid to lose my sight, even though I found the laser painful and uncomfortable I still don’t want to miss my appointments”. (PF14, 52 yrs old)*

***Symptom experience and factors influenced DR*:** Like many conditions of this nature, DR can occur without any initial symptoms and without pain. A noticeable effect on the vision does not typically occur until the disease advances. Symptoms might only be identified once the disease advances, but the typical symptoms of retinopathy to look out for include, sudden changes in vision/blurred vision, eye floaters and spots, double vision and eye pain.

Overall, blurry vision at both near and distance, double vision, seeing floaters, seeing of black spots and glare were the most common symptoms which patients had experienced whereas some patients haven’t experienced any symptoms and signs of DR. Most of the patients’ main complaints was blurry vision. Others stated that seeing of floaters, dark spots and double vision were other symptoms they were experienced. However, the absent of DR symptoms (asymptomatic) was one of the barriers patients reported which delayed seeking help from the eye clinic. One patient voiced on dark spots symptom;

*“It was like a cloud moving in front of my view, I was looking inside the water to let it go but didn’t go away”. (PF6,58 yrs old)*

And another patient commented on double vision says;

*“The post of the building was double and bending when I looked at it” (PM3, 39 yrs old).* Also, a female patient stated that she was uncomfortable when looking under the sun... *“The bright light from the sun caused my vision to dark”. (PF11, 47 yrs old)*

Furthermore, patients were found related their improper diet with their increased blood sugar level and worsening of DR symptoms. Majority blamed that eating rice was significantly increased their blood sugar level. Few commented on eating sweet food were also increased their blood sugar level and affected their vision. One patient commented about rice;

*“I knew that when I ate rice my blood sugar will getting high and my vision also blurry, I tried not to eat rice everyday. (PM4,46 yrs old)*

However, patients concluded that strict glucose control, lifestyle modification, compliance to DR treatments and followed advices from HCWs were the only way to subsize the progression of DR.

***Possible impacts on daily activities*:** It was found that moderate and severe stage of DR have significant impact to health relative quality of life. According to 15 patients, they stated that the most affected activities caused by DR include, reading, driving, cooking, sewing, plumping, mechanic, and social interaction with friends. However, for those patients who had not experienced other complications of DR, the threat of vision loss was the most devastating.

According to 3 school teachers (patients) they mentioned that the need to see well especially when reading and the need for special glasses to assist them in reading and preparing their lecture was the most difficult experienced, they cannot imagine. Patient’s responses as followed;

*“It was very hard for me to prepare my teaching lessons especially when it comes to the marking”. (PM3, 39 yrs old).* Another one says *“I put the laptop close to my eyes when I read, it’s very frustrating.” (PF14, 53 yrs old*) and the last one spoking, *“I have spectacles but my number is keep changing very fast”. (PM8, 52 yrs old)*

Few patients stated that loss of independence, especially mobility and increased fear of accidents had a profound impact on social activities. The majority of patients were no longer did heavy tasks at home and just waited for their family to do things for them. One patient mentioned about driving;

*“I cannot drive my own car, and it very disappointing because when I want to go somewhere, I need someone to drive me, and if no one available I have to wait and did nothing.... sometimes I missed my appointment because no one can drive me to the hospital...”. (PF9, 53 yrs old)*

Also losing of independence resulted from visual impairment was also another devastating for those experienced.

*“Diabetes was affected me so much, now I could not go anywhere, having my own baths, getting my own food and anything else that I want to do.” (PM1, 52 yrs old)*

Other piercing stories obtained from patients include, losing jobs and other important activities that they used to earn money to support their family. One patient stated;

*“I will no longer make smocking for sell and selling of foods as I was afraid to do the cooking and my eyes were so blurry when do the sewing....so we couldn’t support our family anymore even from the little things”. (PF2, 68 yrs old, PF11,47 yrs old)*

Overall, it was found that the impact of visual impairment resulted from DR was conspicuous in patients’ thoughts, activities and lives and also truly affected every individual’s quality of life particularly people living with diabetes.

#### Theme 2: Perceptions toward DR management.

Theme 2 constitutes three subthemes including believe about DR treatment, herbal medicines, and doctor-Patient relationship.

***Believe about DR treatment*:** Over the past years until now, laser therapy and anti-VEGEF injection can substantially improve vision outcomes for patients with clinically significant macular oedema. Overall, this study had discovered that almost patients were just becoming aware of DR treatments include laser and anti-VEGEF injection during their first consultation with ophthalmologist. One patient spoke;

*“I just knew that diabetic retinopathy has various types and also aware that it has treatments (laser and injection). If I’m not come to the clinic, I didn’t hear from the doctor about the treatment of diabetic retinopathy” (PM3, 39 yrs old).*

The results also presented that many patients were having high expectation that laser and injection could bring back their vision to normal. They compared DR with other eye conditions such as cataract and the need for spectacles. It was also clear from the interview that patients were having no choice to decide for their treatments but they just followed of what the ophthalmologist decided for them. One patient spoking;

*“I did not know what the laser and injection did but I trust the doctors when she told me to have either laser or injection because they knew what they doing and they were very intelligence” (PM6, 43 yrs old)*

Majority of patients were satisfied about the treatments they were given, while few people were not satisfied and they were asking for other alternatives like overseas treatments, visiting team from overseas and more effective and powerful laser or injections. One patient asking for overseas treatments;

*“Do we have a chance to referred to overseas for treatment?......I was heard that there are many specialized doctors they can help with retinopathy diseases......”. (PF7,42 yrs old)*

In addition, some patients were perceived that anti-VEGEF injection was better than laser therapy since it was less painful, and cleared their vision more. Responses provided by some patients;

*“I had completed 3 sessions for laser and injections and I feel that injection was better.... there were no more dark spots blocking my vision on my right eye but my left eye was still no improvement despite many lasers” (PF5, 59 yrs old).*

Another participant stated that;

*“I was treating my injection sessions more important because it helped me a lots compared to laser” (PF14, 53 yrs old)*

Conversely, patients with timing laser treatments were found the treatment very effective however, it was very painful and discomforting. One patient voiced;

*“The laser was good to me; it helped my vision to stable but I was scared of it because it very painful.... but I tried to ignore the pain as I love to see better”. (PF10, 61 yrs old)*

***Herbal medicines*:** Over the last few years, herbal medicine was a popular medicine where many people have found to use them to treat their chronic illness include diabetes. However, many studies have found no evidence that herbal medicine could cured diabetes or diabetic retinopathy [20].

According to the interview with patients, it was found that the used of herbal medicines in Kiribati for the treatment of diabetes or DR was not prevalent in most of the participants. The majority reported that they haven’t found yet traditional medicines or herbal medicines for retinopathy. Some patient presented that they only used the non-juice as vitamin substitute but they have no prove that it could lower their sugar level. One patient commented;

*“I used the non-juice for my vitamins because buying veggies were too expensive”. (PF10,58 yrs old).*

However, it was cleared that using of herbal medicines for the treatment of DR was not a common thing in most patients. They believed that laser and injection were most possible treatment for DR but need lots of time and patience.

***Doctor-patients’ relationships*:** Interview results showed that majority of patients were found satisfied with the services provided by the HCWs. Several patients commented on positive interaction with doctors during their previous visits and treatments and others were reported on their negative experiences with doctors. One of the patients commented on HCWs;

*“The doctors and nurses were very kind and helpful, they always give advice to have good control of our diabetes and encouraged us to attend our appointments”. (PF1,41 yrs old)*.

Another patient voiced on the good experience with doctors;

” *The feedbacks from the doctor encouraged me to compliance to my treatments and to control my blood sugar,”. (PM13, 43 yrs old)*.

Also, another patient was commented on negative experienced with doctors;

*“Sometimes doctors were not talked nicely.... maybe they were tired because they have seen lots of patients.... I noticed sometimes they were rushed to see patients and they have no time to explain things......I just came in March and then I didn’t come back because I wanted my bad eye to be treated first but the doctor treated the good one first and I don’t know why... (PF9, 52 yrs old)*

#### Theme 3: Perceived barriers and challenges to diabetic eye care.

Three subthemes formed this theme including education and Knowledge about DR, health care system factors, and access to the services.

***Education and knowledge about DR*:** Most patients during an interview say that they have received enough education about DR given from the doctor and nurses during their diagnosis procedure. However, the transcripts codes indicated that there was, still limited knowledge about DR. Majority just knew that DR was a diabetic eye disease but they did not know how it cause blindness. Many were don’t know that floaters and dot spots were important symptoms of severe DR. Others blamed that lack of education received from HCWs on DR was another reason for not prioritizing their vision. While others say that the eye medical terms were difficult to remember and they recommended HCWs to use posters or eye models when explained about the eye conditions.

One patient response says;

*“The doctors and nurses had explained to me about DR and its urgent symptoms, but I cannot remember because the words are complicated”. (PM3,39 yrs old)*

***Access to the service*:** Lack of access to eye care service is another common problem mentioned by patients. The results shows that people who were living close to the hospital were having no complaints about their access to the service. However, many patients reported that means of transport and dispersion of the islands were the most important barriers to access the eyecare services. Several patients from far places commented that sometimes they did not turn up to their appointment because they found it hard to get on the public transports. Others cited that travelling in longer distances or residing in rural communities were the most challenging for many people to access the eye care services at the time of need. They also added that attending of the eye care services was also costly for them as they need to travel for long distance. One patient spoking about transport inconvenience;

*“Most of the time I have waited for the bus at the road for almost an hour and I just got the chance to get on the public transport......sometimes I got tired and returned home”. (PF6,66 yrs old)*

Another patient commented on the cost of travel;

*“It cost me a lots to access the eyecare services as I have many travels to do, I was living at one of the islets in Abemama (one of the islands) and I have to travel by boat to the main land and from the main land I have to take the plane to reach the eye clinic. It was very expensive but we don’t have choices and most importantly I need to get helped from the eye clinic”. (PM14,56 yrs old)*

***Health care system factors*:** The results presented that majority of patients were complaining about health challenges such as poor referral system, treatment out of stock/medications, length of consultations, missing patient’s folders and lack trained ophthalmologist. One patient commented on the issue with missing folders;

*“Sometimes I came in the morning with less patients but I was becoming last to be seen by the doctor because I don’t have my folder....it could take more than an hour for HCWs to look for our cards.......it very frustrating and disappointing”. (PF9, 52 yrs old)*

Another patient also spoking about referral system;

*“I was working on outer island, and sometimes I need urgent referral to the eye clinic especially when I feel my vision down however, it took time for the nurse to obtain the doctor’s consent for my travel, I wish there is another way that referral from outer island could improve therefore we can get helped as soon as possible” (PM8, 53 yrs old)*

Other patients were also commented on the medication availability and poor health education from HCWs during consultations. One patient voiced;

*“The problem with medication out of stock was affected us......“our diabetes cannot improve if the hospital couldn’t manage to sustain its stock especially for diabetes medications. (PF10,58 yrs old)*

However, these barriers and challenges were associated with the financial constraints within the ministry.

#### Theme 4: Recommendations to improve DR management.

This theme has supported by 3 sub themes include, resources, advances treatments and the need for more regular awareness.

***Resources*:** Most of patients were cited that the need for more ophthalmologist was the most important thing that the healthcare system should consider. They believed that more professional healthcare workers could help to share the work load and provide more quality and efficient outcome to the patients. Others reported that providing more educational materials like eye posters, eye models were very helpful to patients to easily understand about DR and especially their personal condition. One patient spoking

*“Government should support more trainings for HCWs to upgrade their knowledge therefore we don’t need help from overseas”. (PM14,56 yrs old)*

***Advanced treatments*:** It was found from the interview that patients with severe DR condition were that most desperate to seek for more advanced treatments compared to those with mild condition. Few patients perceived that the treatment that were given were not really effective and they recommended to send overseas for treatment. Others were commented on the need for more eye teams from overseas to come so they can have the chance to check their eyes. In addition, several patients conveyed that treatment overseas was beneficial for them in fact there were more specialized doctors that could help the problem with retina. One patient spoking;

“*Is there anything else can be done here.... beside laser and injection? I was worried because I noticed little improvement with my vision.......and I had heard, that there are doctors in overseas which are more specialized on the blood vessels in the eye.... can we have the chance to be sent to overseas?” (PF7, 42 yrs old)*

***Routine awareness*:** Despite routine awareness and regular DR outreach in Kiribati, people’s awareness and understanding about the disease still not common. Suggestions on increasing educational opportunities/awareness of eye care especially on DR were the most frequent response obtained from interviewees. According to some patients they suggested that DR awareness in primary schools could help children to get familiar and understand about DR in their younger age. Other patients recommended that health education during diagnosis procedure and the use of non-medical term helped them to understand more about DR. Few were added that eye model or posters were more easily to understand about DR. Moreover, some patients suggested that community awareness, patients’ participations and ministerial tour were other successful ways to increase peoples’ participation in DR screening. Further suggestions include the availability of the ophthalmologist to see patients on their follow-up/review. One patient voiced;

*“I am an English teacher in one of tertiary level and honestly, I didn’t know about DR, just knew when I got it......now I became an advocator for DR in my work place”. (PM3, 39 yrs old*)

## Discussion

The information gained from patient interviews was crucial to fill in the gaps towards DR management in Kiribati. Perceptions on DR management examined through face-to-face in-depth interviews with twenty-seven DR patients. The findings were resulted into four main themes include the awareness of DR, perceptions toward DR management, perceived barriers and challenges to diabetic eye care, and recommendations to improve DR management.

### Awareness of diabetic retinopathy

Knowledge and awareness were the two important terms used to explain the patient’s level of understanding of DR. Previous studies mentioned the importance of understanding these words, such as awareness of DR was referred to patients when they had heard of DR and knowledge referred to their understanding about DR. Knowledge was crucial as it influenced patients’ attitude and practice patterns toward DR [21].

The results showed that patients were aware that diabetes could affect the eyes and did not know that it could cause irreversible blindness. A cross-sectional study conducted among 377 DM patients in Saudi Arabia showed that patients were also aware of DR and patients’ perceptions of doctors’ advice on DR, the practice of going to check their eyes, and the experience of having their vision affected by DR, were significantly related to their knowledge and awareness of DR [22]. Tajunisah conducted a study in Malaysia among 137 T2DM patients on their first visit to the eye clinic and found that patients attending tertiary-level education were aware of DR complications [23]. Blurry vision was the common symptom of DR, were patients experienced in every stage. The results stated that most patients were lack of awareness of other DR severe symptoms. Most of them mentioned that they had experienced severe DR symptoms but were not concerned about it. Beaser in their mixed methods study conducted online and involved patients with DM only and DM with DR, demonstrated that most of the participants before training were not aware that DR is related to DM and recalled in their interview that they had experienced some severe symptoms of DR but ignored that such symptoms were parts of diabetes [24].

The results also indicated that participants have a good understanding of the possible outcome of DR complications. They mentioned that DR was affecting their well-being, such as loss of self-independence, loss of employment, isolation, stress, and depression. Fenwick cross-sectional study conducted in Melbourne, Australia, among 557 diabetes patients also shared similar impacts of DR that most DM patients had experienced, including difficulty in driving, forgone of many quality life aspects such as work, loss of independence, fear of accidents, and poor social relationship [25]. Knowledge, Attitude, and Practices (KAP) study carried out in Yueqing hospital in Central China among adult diabetic patients found that, since many DM patients were aware of DR, their main problem was social-economic [26].

This study also identified that patients with better education and moderate to severe DR were more adhered to their follow-up clinics and strict glycemic control. A cross-sectional and hospital-based study in India interviewed 376 DR patients, demonstrated that DM patients with better education and attending private hospitals, was found to have a correct awareness of glycaemic control [27].

### Experiences toward DR management

Routine awareness and health education about DR were crucial for patients to avoid misconceptions about DR and other eye conditions. It will also increase patients’ adherence to treatment appointments and follow-up schedules.

The findings demonstrated that patients become aware that DR has alternative treatments, such as laser and injection on their first eye checkup. Some expect that their blindness (DR blindness), could be reversed with laser and injection, just like other eye conditions. A qualitative study conducted on 29 DM patients aged 18–34 in the United Kingdom illustrated that patients did not understand the reason for DR screening and also did not know the treatments when detected with DR [28].

The results also showed that most DR patients had experienced discomfort, fear, and pain from the laser. Some commented that sometimes they did not complete their sessions because their eyes were painful, and the doctor rescheduled them for another time. In addition, Avastin injection was recommended more over the laser treatment. Many patients voiced they liked injection more because it was quicker and less painful than laser. Some commented they had clear vision after being given an injection. The recommendations for VEGEF/Avastin injection have supported the findings from the KAP study in Southwestern Ethiopia [29].

DR patients with moderate-severe DR experienced negative impacts of DR. Some patients mentioned that fear of getting injured, especially when mobilizing, increased their dependence on their family members. For example, one lady commented that she never drives her own car and relies on her family members. The following experiences supported the study results on the experienced of DR patients in Northern Ireland [30].

It also found that most DR patients liked bevacizumab injection more than laser because it was quicker and less painful. Apart from the standard treatments of DR (laser and injection) herbal medicine was found practiced in some parts of the world and not in Kiribati. The findings demonstrated that patients were using herbal medicine as their vitamin substitution. Zhang conveyed that there was no conclusive evidence to prove that single herbal medicine has improved DR regression [31]. In contrast, a study in China has found in their clinical trials that traditional Chinese medicine for DR has improved visual acuity, micro-aneurysms, and HBIAC in DR patients [32].

### Perceived barriers and challenges to received eye care

Poor education was identified as a challenge between patients and HCWs in the working place. The results indicated that patients had received some form of education about DR, but the problem was related to their low education. The evidence showed that most patients preferred eye posters or eye models to communicate their DR conditions.

A qualitative study done in New Orleans highlighted patients believed, that DM education was adequate, but there is a gap between patients’ education provided and their understanding [33].

Transport inconvenience, cost of travel, and the islands’ geography were common barriers mentioned by patients. Patients in rural areas found it difficult and costly to reach eyecare services as they have many travels to cover. Some patients with severe DR commented that the referral procedure was time-consuming because it required many processes. However, some patients have paid their own cost to reach the clinic. Lius’ qualitative study findings conducted in the United States has provided similar results to this current study, including long travel distances to obtain care, limited access to and infrequent use of healthcare, and financial trade-off influenced patients’ adherence to diabetic eye screening [34]. Lu also provided similar results on transport inconvenient in Los Angeles [35].

The study findings also demonstrated that healthcare system failure was the common theme, affecting the patients’ compliance with DR screening. Some patients say that poor hospital record was one of the barriers that caused the delay in running the clinic. A few patients reported that sometimes they missed their clinic because they were tired of waiting, especially when their folder was missing. The results also showed that the lack of ophthalmologists was another contributing barrier to DR screening. People’s expectation to be seen by an ophthalmologist when they visited the hospital was high. Some patients say that they were satisfied when the ophthalmologist saw them and explained their eye conditions. The current findings of this study have also supported the study findings conducted in the United Kingdom on the barriers and facilitators for access to DR screening services [36].

### Suggestions to improve DR management

The results demonstrated that patients were not fully satisfied with DR management and treatment. Most of them were demanding more effective DR treatments. The findings showed common themes, including human resources, advanced treatments, and routine awareness. The need to increase staff was an utmost in the health care system to meet the people’s demands. Patients recommended that increasing the number of ophthalmologists was very important to increase the workflow for eye care and the attendance to DR screening. The study done by Pons supported the findings of this study on the need for more ophthalmologists and reported that more professional doctors will less the waiting time of patients, increase the workflow, and provide quality eye care [37].

The patient’s recommendation for more advanced treatments for DR was useful to consider. It was a bit confusing when patients talked about advanced treatments for DR since laser and anti-VEGEF injections were the latest known treatments commonly used worldwide. The findings showed that patients with severe DR suggested that overseas treatments will give them chances to see better, as they believed that more options could be done overseas. A study conducted in Germany has similar findings to this current study that patients were also not responding well to the current DR treatments and suggested more effective treatments. However, there only a few options could be found after the study on DR etiology and pathology [38].

The results also showed that most of the patients commented on the lack of proper health education and awareness of DR. They said that nurses and doctors have no time to educate them about DR, because of the many patients to see. However, they suggested that HCWs should use social media such as Facebook, and television for DR awareness. They recommended also that the involvement of DR patients in DR campaigns will also increase the number of DM patients attending DR screening. Another important suggestion was the application of eye posters and models to educate patients about DR as they found it easier to understand. This study’s findings were in conjunction with Beaser’s study findings on the strategies to improve the prevention and management of diabetic retinopathy [24].

### Study limitations

This study aimed to explore the perceptions of DR patients on the management of DR in Kiribati. However, the study was only included DR patients in urban settings and failed to explore other crucial barriers for patients in rural areas that may differ from patients in modern settings. Another important limitation included patients who were non-attendees to DR screening had less chance to participate in this study.

## Conclusion

The findings of this study indicated that perception on DR management in Kiribati among DR patients was differed. Majority commented that approaching of the eye care services was a barrier due to the long distances to be undertaken. Lack of awareness and understanding of DR treatments and management were a common barrier found in most non-adherence patients to DR review and treatments. The findings of this study offer implications that can be considered by patients, HCWs, policymakers, and key stakeholders, particularly the Ministry of Health in Kiribati. Patients need to commit to their treatment plans, adhere to prescribed medications, regularly visit health centres, and receive timely treatments. Participation in training sessions is essential for patients to learn from HCWs. Additionally, they must follow their medication regimen closely and report any issues related to DR symptoms to health centres. While healthcare workers maintain good relationships with patients, they should enhance service delivery and create more opportunities for patient education. The Ministry of Health plays a crucial role in supporting patients, families, and the services provided to them. They should bring in trained ophthalmology specialists, replace general doctors with specialists, and train nurses and healthcare workers to specialize in DR care. The Ministry must also ensure that patients have access to adequate medications and work to introduce advanced treatments in Kiribati, in collaboration with other countries in the region. A community-wide campaign focused on preventing DR is needed to help educate people on prevention strategies. This campaign should also promote opportunities for physical activity and provide access to affordable, healthy foods to prevent diabetes.

## Supporting information

S1 Checklist Inclusivity in global research. (DOCX)
